# Production of 11α‐hydroxysteroids from sterols in a single fermentation step by *Mycolicibacterium smegmatis*

**DOI:** 10.1111/1751-7915.13735

**Published:** 2021-03-04

**Authors:** Carmen Felpeto‐Santero, Beatriz Galán, José Luis García

**Affiliations:** ^1^ Centro de Investigaciones Biológicas Margarita Salas Agencia del Consejo Superior de Investigaciones Científicas Madrid Spain

## Abstract

11α‐hydroxylated steroid synthons are one of the most important commercially pharmaceutical intermediates used for the production of contraceptive drugs and glucocorticoids. These compounds are currently produced by biotransformation using fungal strains in two sequential fermentation steps. In this work, we have developed by a rational design new recombinant bacteria able to produce 11α‐hydroxylated synthons in a single fermentation step using cholesterol (CHO) or phytosterols (PHYTO) as feedstock. We have designed a synthetic operon expressing the 11α‐hydroxylating enzymes from the fungus *Rhizopus oryzae* that was cloned into engineered mutant strains of *Mycolicibacterium smegmatis* that were previously created to produce 4‐androstene‐3,17‐dione (AD), 1,4‐androstadiene‐3,17‐dione (ADD) from sterols. The introduction of the fungal synthetic operon in these modified bacterial chassis has allowed producing for the first time 11αOH‐AD and 11αOH‐ADD with high yields directly from sterols in a single fermentation step. Remarkably, the enzymes of sterol catabolic pathway from *M. smegmatis* recognized the 11α‐hydroxylated intermediates as alternative substrates and were able to efficiently funnel sterols to the desired hydroxylated end‐products.

## Graphical abstract

In this work we have rationally designed new recombinant bacteria able to produce 11alpha‐hydroxylated steroids in a single fermentation step using sterols as feedstock. The key elements to achieve this goal were the hydroxylase system from *R. oryzae* and the actinobacteria *Mycocilibacterium smegmatis*.

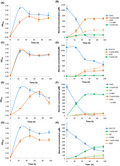

## Introduction

Steroids represent one of the most widely used drugs for multiple clinical purposes (e.g. anti‐inflammatory, immunosuppressive, anti‐allergic, anti‐cancer) (Fernández‐Cabezón *et al*., 2018). The oxidation state of steroid rings and the presence of different attached functional groups determine the specific physiological function of each steroid (Lednicer, 2011), and therefore, structural modifications of steroids, such as hydroxylation, dehydrogenation or esterification, highly affect their biological activity (Donova and Egorova, 2012; Szaleniec *et al*., 2018). Among them, hydroxylation is one of the most important steroid modifications introduced by the pharmaceutical industry, since it introduces deep changes not only in their clinical activities, but also in their physicochemical properties (e.g. solubility, adsorption).

Fungi have been reported to carry out hydroxylations at almost all stereogenic centres of the steroid molecule (Kristan and Rižner, 2012), and they have been widely applied at industrial scale for the production of hydroxylated steroids with a broad range of biological activities (Donova, 2017). This oxyfunctionalization of steroids is mainly catalysed by cytochromes P450 (CYPs) acting as monooxygenases by inserting a single oxygen atom into a non‐activated C–H bond of the substrate with a concomitant reduction of other oxygen atom to water (Bernhardt, 2006). Fungal hydroxylations are usually carried out by two‐component enzyme systems consisting of a CYP monooxygenase and a NAD(P)H CYP‐reductase (CPR) (Crešnar and Petrič, 2011; Kristan and Rižner, 2012).

In particular, 11α‐hydroxylated steroid synthons are one of the most important commercially steroid intermediates used for the production of contraceptive drugs and glucocorticoids. Important fungal species for steroid 11α‐hydroxylation reactions include *Rhizopus nigricans*, *Aspergillus orchraceus*, *Aspergillus niger* and *Rhizopus oryzae* (Wang *et al*., 2017; Kollerov *et al*., 2020). In this sense, the 11α‐hydroxylating system of *R. oryzae* has been used for the production of hydroxyprogesterone (Fernandes *et al*., 2003; Petric *et al*., 2010). This fungal system consists in the CYP509C12, one of the 48 CYPs encoded in its genome, and its redox partner RoCPR1, a NAD(P)H‐dependent CRP. By expressing CYP509C12 in yeast, it has been demonstrated that this CYP hydroxylates predominantly at 11α and 6β positions of steroids (Petric *et al*., 2010), although 11α‐hydroxylation of steroids by fungi is the synthetic procedure currently used by the steroid industry (Petric *et al*., 2010). The current method for obtaining 11α‐OH‐AD/ADD at industrial level is a two‐step one‐pot bioconversion from phytosterols based on the specific biochemical activities of two microbial strains without separation and purification of the intermediate product (Dovbnya *et al*., 2017). However, the large number of CYPs contained in the fungal strains often generates during the *in vivo* hydroxylation a multiplicity of oxygenated steroid by‐products, which not only are difficult to separate, but also contribute to reduce the bioconversion yields (Fernandes *et al*., 2003). Therefore, the design of alternative and more specific hydroxylating microbial cell factories created by recombinant DNA technologies has been considered an industrial challenge (Donova, 2017; Fernández‐Cabezón *et al*., 2018).

In this sense, the cloning and expression of hydroxylating CYPs and their CPRs partners in heterologous hosts to be used as alternative fungal bio‐catalyzers has been explored in yeasts and bacteria (Szczebara *et al*., 2003; Schmitz *et al*., 2014; Schiffer *et al*., 2015; Ichinose *et al*., 2015; Hull *et al*., 2017; Uno *et al*., 2017; Wang *et al*., 2017; Lu *et al*., 2018; Felpeto‐Santero *et al*., 2019). However, all these bio‐catalysers described so far are only able to transform pre‐synthetized steroid synthons, and therefore, the complete synthesis requires two fermentation steps, one to produce the first synthons and other to hydrolyxate them. Up to now, none of the developed hydroxylating recombinant bio‐catalyzers provide the possibility of producing hydroxylated steroids in a single fermentation step starting from natural sterols.

**Fig. 1:**
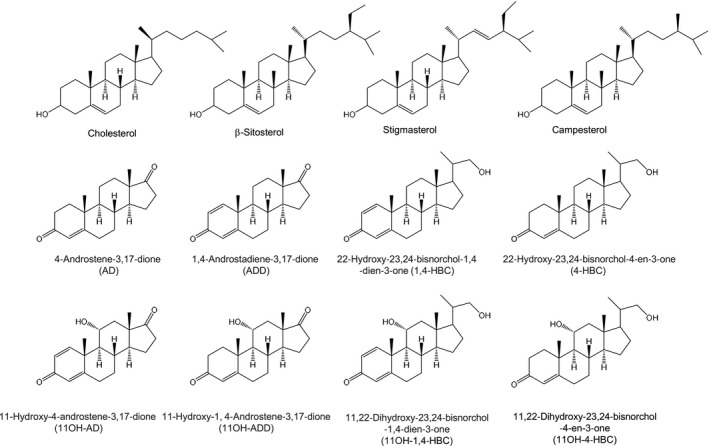
Chemical structure of steroidal compounds used in this work.

Some actinobacteria (e.g. *Mycobacterium*, *Mycolicibacterium*, *Gordonia, Rhodococcus*) that are able to metabolize cholesterol and other natural sterols are currently used by the industry to obtain steroid synthons (*e*.*g*. 4‐androstene‐3,17‐dione (AD), 1,4‐androstadiene‐3,17‐dione (ADD), 9‐hydroxy,4‐androstene‐3,17‐dione (9OH‐AD), testosterone (TS) and C22 steroids (e.g. 22‐hydroxy‐23,24‐bisnorchol‐4‐en‐3‐one (4‐HBC), 3‐oxo‐23,24‐bisnorchol‐1,4‐dien‐22‐oic acid (1,4‐HBC), 9,22‐dihydroxy‐23,24‐bisnorchol‐4‐en‐3‐one (9OH‐4‐HBC) from sterols (phytosterols (PHYTO) or cholesterol (CHO)) (Fig. 1) (Donova and Egorova, 2012; García *et al*., 2012; Galán *et al*., 2017). Although most of the strains used at industrial scale are natural isolated or random mutagenized strains, in the last few years a number of new steroid‐producing strains have been rationally designed by metabolic engineering approaches (Wei *et al*., 2010, 2014; Xiong *et al*., 2017; He *et al*., 2018; Liu *et al*., 2018; Zhang *et al*., 2018). In this way, we have developed several mutant strains of *Mycolicibacterium smegmatis* (formerly *Mycobacterium smegmatis*) able to produce *à la carte* a large number steroid synthons (Galán *et al*., 2017; García et al., 2017).

In this work, we have tested the possibility of rationally design new recombinant bacteria able to produce 11α‐hydroxylated steroids in a single fermentation step using sterols as feedstock. To this aim, we have designed a synthetic operon containing the 11α‐hydroxylating enzymes from *R. oryzae* that was cloned and expressed into two previously engineered mutant strains of *M. smegmatis* created to produce AD or ADD from sterols. The expression of the synthetic operon in these modified bacterial chassis has allowed us to produce for the first time 11α‐hydroxylated compounds directly from sterols in a single fermentation step.

## Results

### Production of 11αOH–ADD in *M. smegmatis*

To test the possibility of producing 11α‐hydroxylated steroids from natural sterols (CHO and PHYTO) in a single step in *M. smegmatis,* we designed the synthetic bacterial operon FUN (RoCPR59830‐CYP509C12), harbouring the CYP 11α‐hydroxylase from *R. oryzae* and its redox partner CRP (Fig. S1). The synthetic operon was cloned into the mycobacterial replicative plasmid pMV261, creating the pMVFUN plasmid, that was transformed into the *M. smegmatis* MS6039 mutant strain. This mutant strain has been previously engineered and accumulates ADD from CHO or PHYTO (Table 1) (García *et al*., 2012; García et al., 2017). The expression of the enzymes RoCPR59830 and CYP509C12 in the MS6039 (pMVFUN) recombinant strain was determined by SDS‐PAGE (Fig. S2A) and the ability to transform sterols into 11αOH‐ADD was analysed by HPLC‐MS along the growth curve using sterols (CHO or PHYTO) as feedstock.

**Table 1:** Strains, plasmids and oligonucleotides used in this work

	Description	References
Strain		
*Mycolicibacterium smegmatis* mc^2^ 155	ept^‐1^, mutant mc^2^6	Snapper *et al*., 1990
*Mycolicibacterium smegmatis* mc^2^ 155 MS6039	*M. smegmatis* mc^2^ 155 Δ*MSMEG_6039*	Galán *et al*., 2017
*Mycolicibacterium smegmatis* mc^2^ 155 MS6039‐5941	*M. smegmatis* mc^2^ 155 Δ*MSMEG_6039‐*Δ*MSMEG_5941*	Galán *et al*., 2017
*Escherichia coli* DH10B	F^‐^, *mcrA, Δ(mrr‐hsdRMS‐mcrBC), f80ΔlacZDM15 ΔlacX74, deoR, recA1, endA1, araD139, Δ(ara‐leu)7697, galU, galK, rpsL, nupG, λ^‐^*	Invitrogen
Plasmid		
pMV261	*Km^R^, Mycobacterium* expression vector, *P_Hsp60_*	Stover *et al*., 1991
pUC57FUN	*Ap^R^,* synthetic operon FUN into *E. coli* cloning vector pUC57	Provided by ATG:biosynthetics GmbH
pMVFUN	*Km^R^*, synthetic operon FUN into pMV261	This work

Oligonucleotide	Sequence	Application
pMV4260 F	TTGCCGTCACCCGGTGACC	pMV261 sequencing
pMV4486 R	ATCACCGCGGCCATGATGG	pMV261 sequencing
RoCPR1 IntF1	TTCAAGATCGAAGAACTGACG	FUN operon sequencing
RoCPR1 IntF2	CGGTGTGAACACCAACTACC	FUN operon sequencing
Cyp509C12 IntF1	CATCTTGAACATCTTCGGTCC	FUN operon sequencing

**Fig. 2:**
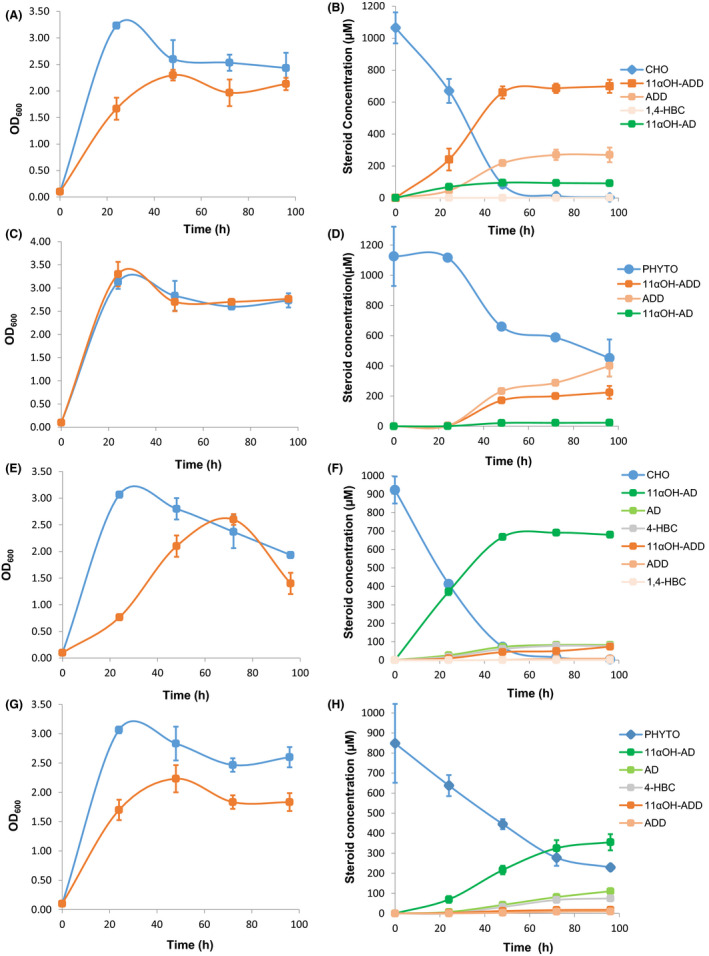
Biotransformation assays in the presence of sterols (CHO or PHYTO) performed with the MS6039 (pMVFUN) and MS6039‐5941 (pMVFUN) recombinant strains. Results represent means of three biological replicates. Error bars represent the standard deviation. A. Growth curves of MS6039 (pMVFUN) (red) and MS6039 (pMV261) (blue) strains in the biotransformation medium containing CHO. B. Consumption of CHO and steroidal biotransformation products delivered by MS6039 (pMVFUN). C. Growth curves of MS6039 (pMVFUN) (red) and MS6039 (pMV261) (blue) in the biotransformation medium containing PHYTO. D. Consumption of PHYTO and steroidal biotransformation products delivered by MS6039 (pMVFUN). E. Growth curves of MS6039‐5941 (pMVFUN) (red) and MS6039‐5941 (pMV261) (blue) strains in the biotransformation medium in the presence of CHO. F. Consumption of CHO and steroidal biotransformation products delivered by MS6039‐5941 (pMVFUN). G. Growth curves of MS6039‐5941 (pMVFUN) (red) and MS6039‐5941 (pMV261) (blue) strains in the biotransformation medium in the presence of PHYTO. H. Consumption of PHYTO and steroidal biotransformation products delivered by MS6039‐5941 (pMVFUN).

First, MS6039 (pMVFUN) and MS6039 (pMV261) strains were grown in the biotransformation medium in the presence of CHO. During the exponential phase, the recombinant strain MS6039 (pMVFUN) showed a slight delay in growth compared to the control strain MS6039 (pMV261), but both cultures reached similar biomass at the stationary phase (Fig. 2A). After 24 h, 11αOH‐ADD was detected in the culture supernatant of MS6039 (pMVFUN) and the maximum bioconversion of 99.6 ± 0.3 % was obtained after 60 h of growth with a production yield of 65.8 ± 3.9 % for φ11αOH‐ADD/CHO (Fig. 2B). Interestingly, the HPLC‐UV/DAD‐MS monitoring allowed us to identify and quantify small amounts of some by‐products in the culture medium, such as ADD, 11αOH‐AD and trace amounts of 1,4‐HBC (Fig. 2B). Production yields for the main by‐products were 25.4 ± 4.3% for ADD/CHO and 8.6 ± 0.5% for 11αOH‐AD/CHO (Fig. 2B, Fig. S3A and B). We have detected one additional compound eluting at 4.92 min (Fig. S3A and B). Its m/z of 345 coincides with the molecular mass of 1,4‐HBC increased by 16, suggesting that it could correspond to 11αOH‐1,4‐HBC.

As expected, MS6039 (pMV261) control strain did not produce 11αOH‐ADD from CHO (Fig. S3A) and ADD was detected as the main biotransformation product with a conversion rate of 96.2 ± 5.9% and a production yield of 99.3 ± 0.3% for ADD/CHO.

Taking into account that PHYTO are used in the steroid industry as the preferred low‐cost raw material to produce steroid synthons, we tested it as feedstock to produce 11αOH‐ADD in the MS6039 (pMVFUN) recombinant strain. To this aim, MS6039 (pMVFUN) and MS6039 (pMV261) strains were grown in the biotransformation medium in the presence of PHYTO and monitored by HPLC DAD‐MS as performed for CHO cultures (Fig. 2C). The MS6039 (pMVFUN) strain successfully achieved the transformation of PHYTO into 11αOH‐ADD. The conversion rate was 67.5 ± 0.3%, and the 11αOH‐ADD production yield 11αOH‐ADD/PHYTO was 33.3 ± 0.2% (Fig. 2D and Fig. S4). Some by‐products as ADD, 11αOH‐AD, 1,4‐HBC and 11αOH‐1,4‐HBC were detected when PHYTO was used as feedstock (Fig. S4). The yields for these by‐products were 59.5 ± 0.4% for ADD/PHYTO and 3.4 ± 0.1% for 11αOH‐AD/PHYTO. Derived compounds 11αOH‐1,4‐HBC and 1,4‐HBC could not be quantified because they were present at very low concentrations.

As expected, MS6039 (pMV261) control strain only produced ADD from PHYTO with a conversion rate of 67.5 ± 0.3% and a transformation yield of 95.7 ± 0.9% for ADD/PHYTO (Fig. S4).

### Production of 11αOH–AD in ***M. smegmatis***

To achieve the conversion of sterols into 11αOH‐AD in a single fermentation step, we used as chassis the *M. smegmatis* MS6039‐5941 mutant strain that was engineered to produce AD from CHO or PHYTO (Table 1) (García *et al*., 2012; García et al., 2017). The MS6039‐5941 strain was transformed with the pMVFUN plasmid as described above. The production of CYP509C12 and RoCPR59830 proteins in the recombinant MS6039‐5941 (pMVFUN) strain was confirmed by SDS‐PAGE (Fig. S2B), and the ability to transform sterols (CHO and PHYTO) into 11αOH‐AD was analysed by HPLC‐MS.

First, MS6039‐5941 (pMVFUN) and control MS6039‐5941 (pMV261) strains were cultured in the biotransformation medium in the presence of CHO. As observed with MS6039 (pMVFUN), during the exponential phase, the recombinant strain MS6039‐5941 (pMVFUN) showed a delay in growth compared to the control strain MS6039‐5941 (pMV261), but this effect was partially restored at stationary phase (Fig. 2E). CHO started to be consumed after 48 h of growth and completely depleted at 96 h. The product yield reached was 74.0 ± 0.2% 11αOH‐AD/CHO (Fig. 2F). Some by‐products as AD, 4‐HBC, 11αOH‐ADD, ADD and 1,4‐HBC were produced during the biotransformation (Fig. 2F, Fig. S5A and B). The more relevant detected by‐products were AD (AD/CHO = 9.0 ± 1.0%), 4‐HBC (4‐HBC/CHO = 8.3 ± 0.4%) and 11αOH‐ADD (11αOH‐ADD/CHO = 7.9 ± 0.4%). We have detected two additional compounds eluting at 2.2 and 5.7 min (Fig. S5A and B). Based on their m/z, they could be assigned to the double hydroxylated compound 11α, 6β‐diOH‐4‐HBC (m/z of 319) and 11αOH‐4‐HBC (m/z of 319) (Fig. S5A and B).

It is worth to mention that the control strain MS6039‐5941 (pMV261) did not produce 11αOH‐AD (Fig. S5) and, as expected, AD is the main product, having a yield of 76.7 ± 3.7% with a conversion of 99.1 ± 0.2%. Curiously, a significant amount of 4‐HBC was detected in the control strain (4‐HBC/CHO = 18.3 ± 2.9 %) when compared to the strain carrying the FUN operon. A small amount of ADD was also detected in the control strain (ADD/CHO = 4.9 ± 0.9%).

On the other hand, the MS6039‐5941 (pMVFUN) strain was grown in the presence of PHYTO to check its ability to convert this raw material into 11αOH‐AD. During the biotransformation, the recombinant strain MS6039‐5941 (pMVFUN) showed also a slight decrease in growth compared to the control strain MS6039‐5941 (pMV261) (Fig. 2G). The HPLC monitoring revealed that this strain transformed PHYTO into 11αOH‐AD (Fig. 2H). As it is shown, phytosterols reached a conversion of 73.0 ± 1.7% at 96 h. At this time, the product yield was 57.3 ± 5.3% for 11αOH‐AD/PHYTO. In addition, other by‐products, such as AD (AD/PHYTO = 17.9 ± 1.3%), 4‐HBC (4‐HBC/PHYTO = 12.1 ± 2.3%), 11αOH‐ADD (11αOH‐ADD/PHYTO = 2.9 ± 0.2%) and ADD (ADD/PHYTO = 1.6 ± 0.5%) were detected in the culture supernatant (Fig. 2H and Fig. S5). As expected, MS6039‐5941 (pMV261) control strain did not render any 11αOH‐AD (Fig. S6) and only produced AD achieving a conversion of 52.7 ± 12.9 % yielding 77.6 ± 2.0 % AD/PHYTO. A significant amount of 4‐HBC (17.0 ± 1.2% 4‐HBC/PHYTO) and a small amount of ADD (ADD/PHYTO = 3.3 ± 0.4%) were produced as by‐products (Fig. S6).

## Discussion

In the pharmaceutical sector, steroid hydroxylation plays an important role to produce new functionalized steroids, because it usually introduces deep changes in their physicochemical and pharmaceutical properties. In particular, the 11α‐ or 11β‐hydroxylation of steroids are essential functionalization steps to develop commercially important intermediates to synthetize glucocorticoids and contraceptive drugs. Current methods of production of hydroxylated steroids mainly rely on biotransformations using wild‐type fungal whole cells that harbour these enzymatic activities. The production of the hydroxylated steroids is carried out in at least two fermentation steps exhibiting in most cases some drawbacks such as low selectivity and reduced conversion yield. Therefore, the design of alternative fermentation processes by using recombinant DNA technologies has been proposed in recent years. In this sense, several fungal hydroxylases have been successfully expressed in yeasts (Petric *et al*., 2010; Hull *et al*., 2017; Lu *et al*., 2018) demonstrating their potential biotechnological applications. However, to the best to our knowledge these recombinant yeasts have not been implemented at industrial scale yet.

In this work, we have advanced one‐step forward in the direction of creating alternative processes to produce the 11‐hydroxylated steroids directly from sterols in a single fermentation step. To fulfil this aim, we have used a rationally designed synthetic operon (named FUN operon) carrying the 11α‐hydroxylating system from *R. oryzae* and cloned them in *M. smegmatis* generating new recombinant bacterial strains able to produce 11‐hydroxylated derivatives in a single fermentation directly from natural sterols such as CHO and PHYTO.

*M. smegmatis* is a model microorganism that has been used for the study of steroid catabolism due to some strengths: (i) it is a fast‐growing bacterium capable to grow in natural sterols (CHO and PHYTO) as a sole carbon and energy source; (ii) its genome sequence has been accessible since 2006; (iii) it can be manipulated genetically with many genetic tools; (iv) it is a robust chassis resistant to stressing industrial production conditions; and (v) it has a very effective transport system for sterols. Hence, *M. smegmatis* has been proposed and used before as an efficient platform to produce steroidal drugs (Fernández‐Cabezón *et al*., 2017; Galán *et al*., 2017).

One of the most important achievements of this work is the demonstration that the synthetic fungal operon was fully functional in *M. smegmatis*. Only few fungal CYPs have been successfully produced in its active form in bacteria so far (Barnes *et al*., 1991; González and Korzekwa, 1995; Hannemann *et al*., 2006; Felpeto‐Santero *et al*., 2019). In our case, we achieved significant levels of CYP and CRP expression as determined by SDS‐PAGE (Fig. S1). Apparently, depending on the substrate and the mutant strain used, the production of the hydroxylating system generates some cellular stress in the host as deduced by comparing the corresponding growth curves (Fig. 2).

The detection of the 11α‐hydroxylated steroids in the culture medium of the recombinant bacteria at high yields demonstrated that the fungal 11α‐hydroxylating enzymes have been integrated in the sterol metabolism in *M. smegmatis* strains creating a new expanded pathway. Such an efficient integration was in fact a surprising result, because one likely outcome could be the hydroxylation of different metabolic intermediates that might block some of the multiple reactions required to accumulate the desired compound due to the specificity of the sterol catabolic enzymes. However, MS6039 (pMVFUN) and MS6039‐5941 (pMVFUN) recombinant strains successfully accumulated 11αOH‐AD and 11αOH‐ADD, respectively, using CHO and PHYTO as precursors. In this sense, the detection of 11αOH derivatives of AD, ADD, 4‐HBC and 1,4‐HBC suggests, on the one hand that the enzymes of *R. oryzae* are not highly specific for a particular steroid. However, on the other hand, this also means that although several hydroxylated intermediates can be produced, none of them blocks the pathway, and fortunately, can be finally funnelled to the final products 11αOH‐AD or 11αOH‐ADD, depending on the mutant host used. Therefore, we can conclude that the enzymes of the *M. smegmatis* sterol degradation pathway can also recognize as substrates the 11αOH derivatives of their own natural substrates.

It is worth to mention that when the 11α‐hydroxylating system of *R. oryzae* was expressed in yeast, it was able to hydroxylate steroids, both at 11α and 6β positions, with good yields (Petric *et al*., 2010). However, in our case the main products obtained were hydroxylated at 11α position. We have detected trace amounts of a compound of m/z 319 compatible with the dihydroxylated derivative of 4‐HBC in the biotransformation of CHO by the MS6039‐5941 (pMVFUN) strain. Although more experiments are required to confirm the presence of small amounts of 6β‐OH derivatives in *M. smegmatis*, this finding suggests that 6β‐hydroxylation is not relevant in our recombinant strain. This interesting result suggests that, most probably, the specificity of CYP enzymes depends on their metabolic environment (location, pH, substrates, other reductases, etc.), and the hydroxyl group destination (11α or 6β position) can be favoured in one selective direction. This characteristic has been also previously observed in the case of heterologous expression of the same *R. oryzae* hydroxylating system in yeast where the distribution between 11α‐ and 6β‐hydroxylated derivatives, and the appearance of non‐identified monohydroxysteroids depended on the substrate that was used (Petric *et al*., 2010).

Although the whole process has to be further optimized at industrial scale to improve the yield of the 11α‐hydroxylated compounds and to reduce the amount and number of by‐products, our results open a new avenue for searching effective biocatalysts for producing hydroxylated steroids from sterols in a single fermentation step. In addition, they reinforce the assumption that engineered *M. smegmatis* strains represent a new generation of biocatalysts with a great potential to be applied for industrial processes. Based on the increased knowledge on the steroid metabolism in *M. smegmatis,* we uphold this bacterium as an exceptional bacterial chassis to implement *à la carte* metabolic engineering strategies based on synthetic biology for the industrial production of other valuable pharmaceutical steroids directly from sterols.

## Experimental procedures

### Chemicals

Commercial phytosterols from pine (provided by Gadea Biopharma, Spain) containing a mixture of different sterols (w/w percentage): β‐sitosterol (83.61 %), stigmasterol (8.79 %) and campesterol (7.59 %), 22‐hydroxy‐23,24‐bisnorchol‐4‐en‐3‐one (4‐HBC > 98 %) and 3‐oxo‐23,24‐bisnorchol‐1,4‐dien‐22‐oic acid (1,4‐HBC > 97 %) were kindly given by Gadea Pharma S.L., 4‐androstene‐3,17‐dione (AD > 99.0%), 1,4‐androstadiene‐3,17‐dione (ADD > 98.0%) and testosterone (TS > 98.0%) were purchased from TCI America. Cholesterol (CHO > 99.0%) and 11α‐hydroxy‐4‐androstene‐3,17‐dione (11αOH‐AD > 98.0%) were purchase from Sigma‐Aldrich.

### Strains, oligonucleotides and culture growth

The strains, plasmids and oligonucleotides used in this study are listed in Table 1. *Escherichia coli* DH10B strain was used as a host for cloning. It was grown in rich LB medium at 37°C in an orbital shaker at 200 rpm. LB agar plates were used for solid media. Gentamicin (10 μg ml^−1^), ampicillin (100 μg ml^−1^) or kanamycin (50 μg ml^−1^) were used for plasmid selection and maintenance in this strain. *M. smegmatis* mc^2^ 155 was cultured on *Bacto Middlebrook* 7H9 (7H9, *Difco)* supplemented with *Middlebrook ADC Enrichement* (ADC, *Difco)* (10 % v/v), glycerol (0.2 % v/v) (*Sigma)* at 37°C in an orbital shaker at 200 r.p.m. Tween 80 % (0.05 % v/v) (*Sigma)* was added to *M. smegmatis* cultures to avoid cell aggregation. Antibiotics were used where indicated at the following concentrations: kanamycin (20 µg ml^‐1^). Cell grow was monitored following OD_600nm_.

### Design and construction of the bacterial synthetic FUN (CYP509C12‐RoCRP1) operon

To achieve the heterologous production of the 11α‐hydroxylase activity from *R. oryzae* RA 99‐880 (Fungal Genetics Stock Center, FGSC, University of Missouri; Petric *et al*., 2010) in bacteria, we designed the synthetic operon FUN that encodes the cytochrome CYP509C12 (EIE80372) with 11α‐hydroxylase activity and its natural redox partner RoCPR1 cytochrome reductase (EIE89541). The codon usage was manually optimized for *M. smegmatis*, keeping a nucleotide identity percentage of approximately 83% for both genes. A consensus Shine–Dalgarno sequence of 6 bp (AAAGGGAG) was added upstream the respective start codons as well as some restriction sites to facilitate cloning (Fig. S1). Alanine was also included as the second amino acid of the resulting proteins to increase protein translation (Bivona *et al*., 2010). The operon engineered was chemically synthesized by ATG:biosynthetics GmbH and initially cloned into the pGH vector yielding plasmid pGH‐FUN that was used to transform *E. coli* DH10B cells and check the sequence. Plasmid pGH‐FUN was digested with *Bam*HI‐*Eco*RI to release the fragment containing the FUN operon that was further cloned under the control of the *P_hps_* constitutive promoter, into the shuttle *E. coli/Mycobacterium* vector pMV261 (Stover *et al*., 1991) yielding pMVFUN. Plasmids pMV261 (empty vector, control plasmid) and pMVFUN were individually transformed into the *M. smegmatis* mutants by electroporation.

### Analysis of the FUN operon expression by SDS‐PAGE analysis

The recombinant strains MS6039 *and* MS6039‐5941 carrying plasmid pMVFUN (Table 1) were grown in biotransformation media and conditions during 24 h. Cells were harvested by centrifugation (15 min, 5000 × *g*, 4 °C) thawed on ice, washed twice with NaCl 0.9 % (w/v) and resuspended in 0.5 ml of Tris‐HCl 50 mM pH 7.5. Cells were disrupted by sonication using a Branson sonicator 150 (6‐8 pulses of 1 min at 90% power, with 30 s of cooling on ice between each). Cell debris was removed by centrifugation at 14,000 × *g* for 15 min at 4 °C. Soluble and insoluble fractions were analysed by SDS‐PAGE to check FUN operon expression.

### Bioinformatic tools

DNA and protein sequences from *R. oryzae* genome were obtained from Broad Institute Server (http://www.broad.mit.edu/annotation/genome/rhizopusoryzae/MultiHome.html) and National Center for Biotechnology Information (NCBI) Database (http://www.ncbi.nlm.nih.gov/gene/). Nucleotide and amino acid sequences were compared to *National Center for Biotechnology Information* (NCBI) database using BLAST algorisms (http://www.ncbi.nlm.nih.gov/cgibin/Entrez/genom_table_cgi); to align and compared to local database, Local‐BLAST (BioEdit) and HMMER algorithm at *European Bioinformatics Institute* (EBI) (http://www.ebi.ac.uk/Tools/hmmer/) were used. Multiple alignments of protein were carried out with the MUSCLE server programme at the European Bioinformatics Institute (EBI) (http://www.ebi.ac.uk/Tools/msa/muscle/).

### Steroid biotransformation assay

Sterol biotransformation assays were carried out using *M. smegmatis* MS6039 (pMVFUN) and MS6039‐5941 (pMVFUN) growing cells in shaken 100 ml flasks containing 20 ml of biotransformation medium having the following composition: 7H9 Broth supplemented with 18 mM glycerol as starter, 1 mM cholesterol or 1 mM phytosterols (previously dissolved in 3.6 % (v/v) Tyloxapol), 0.5 mM δ‐aminolevulinic acid (ALA) and kanamycin (20 µg ml^‐1^). First, a well‐grown (48h) pre‐culture of the mycobacterial recombinant strain cultured in Bacto Middlebrook 7H9 (7H9; Becton, Dickinson and Company, Franklin Lakes, NJ, USA) supplemented with Middlebrook ADC Enrichement (ADC, Difco) (10 % v/v), glycerol (0.2 % v/v) (Merck Life Science S.L.U., Madrid, Spain), kanamycin (20 µg ml^‐1^) and Tween 80 % (0.05 % v/v) (Merck Life Science S.L.U.) added to avoid cell aggregation. The pre‐culture was centrifuged and washed with one volume of NaCl‐Tween 80 solution prior to its inoculation. The pellet was resuspended in 0.5 ml of the washing solution to measure the OD_600_. The biotransformation flasks were inoculated to an initial OD_600_ of 0.05 and cultured on an orbital shaker (250 rpm) at 37°C during 96 h. Culture samples (1 ml) were taken every 24 h (0, 24, 48, 72 and 96 h) to monitor growing (OD600) and sterol modifications (HPLC‐DAD‐MS).

### Steroids extraction

Aliquots of 10 µl of 5 mM TES in 10 % (v/v) tyloxapol were added to each 0.2 ml sample taken from the biotransformation experiments prior to extraction with chloroform, as an internal standard (ISTD). The samples were extracted using two volumes of chloroform. The aqueous fraction was discarded, and the chloroform fraction was dried at 60 °C using a Thermoblock and then dissolved in 0.5 ml of acetonitrile. Each sample was subjected to chromatographic analysis by HPLC‐UV/DAD‐MS (25 µl).

### HPLC‐UV/DAD‐MS analysis

Experiments were carried out using a DAD detector and a LXQ Ion Trap Mass Spectrometer, equipped with an atmospheric pressure chemical ionization source, electrospray ionization source and interfaced to a Surveyor Plus LC system (all from Thermo Electron, San Jose, CA, USA). Data were acquired with a Surveyor Autosampler and MS Pump and analysed with the Xcalibur Software (from Thermo Fisher Scientific, San Jose, CA, USA). High‐purity nitrogen was used as nebulizer, sheath and auxiliary gas. MS analysis was performed both in full scan and in selected ion monitoring (SIM) mode by scanning all the daughter ions of the products in positive ionization mode. The quantification was performed from parent mass of compounds, and the specificity was obtained by following the specific fragmentations of all compounds.

The experiments were carried out with the following interface parameters: Ionization source APCI, volume to inject 25 µl, capillary temperature 275°C, vaporizing temperature 425°C, capillary voltage 39 V, corona discharge 6 kV, source power 6 µA and dissociation by collision energy 15 eV. Use high pure nitrogen as an auxiliary gas and sprayer. Chromatographic separation was performed on a *Tracer Excel* 120 ODSB C18 (4.6 mm x 150 mm, particle size 5 μm) (Teknokroma). The chromatography was performed using water containing 0.1 % (v/v) of formic acid, acetonitrile containing 0.1 % (v/v) of formic acid and isopropanol containing 0.1 % (v/v) of formic acid as mobile phases A, B and C, respectively (flow 1 ml min^‐1^). To monitor cholesterol (CHO) biotransformation, the examined compounds were as follows: TES (289 m/z) used as ISTD (internal standard): CHO (269 m/z), AD (287 m/z), 11αOH‐AD (303 m/z), ADD (285 m/z), 11αOH‐ADD (301 m/z), 4‐HBC (331 m/z) and 1,4‐HBC (329 m/z). The HPLC gradient used was as follows:

**Table:** 

Time (min)	% A	% B	% C
0	50	50	0
5	50	50	0
15	20	71	9
20	4	87	9
40	0	85	15
41	0	85	15
42	50	50	0
52	50	50	0

Quantification was performed by an ISTD (Internal Standard) method. The quantification of the compounds was calculated by the reaction yield and peak area regarding to IS. To monitor the biotransformation of PHYTO, that is a mixture of sitosterol (SITO), stigmasterol (STIG) and campesterol (CAMP), the examined compounds were as follows: TES (289 m/z) used as ISTD; CAMP (383 m/z), STIG (395 m/z); SITO (397 m/z), AD (287 m/z), 14αOH‐AD (303 m/z), 11αOH‐AD (303 m/z), ADD (285 m/z), 11αOH‐ADD (301 m/z), 4‐HBC (331 m/z) and 1,4‐HBC (329 m/z). The HPLC gradient used was as follows:

**Table:** 

Time (min)	% A	% B	% C
0	50	50	0
5	50	50	0
15	20	71	9
20	0	91	9
40	0	70	30
41	0	85	15
42	50	50	0
52	50	50	0

The concentration of sterols in 1 mM PHYTO is as follows: 0.84 mM SITO + 0.08 mM STIG + 0.08 mM CAMP. Therefore, the quantity of PHYTO represents the sum of the quantities of SITO, STIG and CAMP. The quantification of the compounds was calculated by the reaction yield and peak area regarding to IS.

## Conflict of interest

None declared.

## Supporting information


**Fig. S1**. Schematic representation of the genes contained in FUN operon (RoCPR1‐CYP509C12). The sequences of the intergenic regions R1‐R3 are indicated in the table. The sequence of restriction sites is underlined and RBS sequences are indicated in bold. The main restriction sites in the synthetic operon are also indicated.
**Fig. S2**. SDS‐PAGE analysis of the 11α hydroxylating enzymes from *R. oryzae* in the *M. smegmatis* recombinant strains. (A) Lanes 1 and 6, molecular mass markers; lane 2, soluble (SN) control extract from MS6039 (pMV261); lane 3, soluble extract from MS6039 (pMFUN); lane 4, insoluble fraction from MS6039 (pMV261) and lane 5, insoluble fraction from MS6039 (pMVFUN). (B) Lanes 1 and 6, molecular mass markers; lane 2, soluble (SN) control extract from MS6039‐5941 (pMV261); lane 3, soluble extract from MS6039‐5941 (pMFUN); lane 4, insoluble fraction (P) from MS6039‐5941 (pMV261) and lane 5, insoluble fraction from MS6039‐5941 (pMVFUN). The bands of CYP and CPR proteins are indicated by asterisks and arrows.
**Fig. S3**. CHO biotransformation by MS6039 (pMVFUN) (FUN) and MS6039 (pMV261) (Control) strains. A) HPLC‐DAD chromatogram (50‐600 nm) (green) and full scan mass spectra (m/z 150‐400) at 0 h and 96 h of growth are shown. B) Magnification of the first 14 min of the HPLC‐DAD chromatogram (50‐600 nm) green, full scan mass spectra (m/z 150‐400), mass spectra of m/z 301 and m/z 303 corresponding to 11αOH‐ADD and 11αOH‐AD are shown
**Fig. S4**. PHYTO biotransformation by MS6039 (pMVFUN) (FUN) strain. HPLC‐DAD chromatogram (50‐600 nm) (green), full scan mass spectra (m/z 150‐400), mass spectra of SITO (m/z 387), CAMP (m/z 383), STIG (m/z 386), 11αOH‐ADD (m/z 301) and ADD (m/z 286) at 96 h are shown.
**Fig. S5**. CHO biotransformation by MS6039‐5941 (pMVFUN) (FUN) and MS6039‐5941 (pMV261) (Control) strains. A) HPLC‐DAD chromatogram (50‐600 nm) (green) and full scan mass spectra (m/z 150‐400) at 0 h and 96 h of growth are shown. B) Magnification of the first 20 min of the HPLC‐DAD chromatogram (50‐600 nm) green, full scan mass spectra (m/z 150‐400), mass spectra of m/z 303 corresponding to 11αOH‐AD are shown..
**Fig. S6**. PHYTO biotransformation by MS6039‐5941 (pMVFUN) (FUN) strain. HPLC‐DAD chromatogram (50‐600 nm) (green), full scan mass spectra (m/z 150‐400), mass spectra of residual SITO (m/z 387), CAMP (m/z 383), STIG (m/z 386) and 11αOH‐AD (m/z 303) at 96 h of growth are shown.
