# Supplementary material for: Production of 11α‐hydroxysteroids from sterols in a single fermentation step by Mycolicibacterium smegmatis
Source: Microb Biotechnol. 2021 Mar 4;14(6):2514–24. doi: 10.1111/1751-7915.13735 (PMC8601193; doi:10.1111/1751-7915.13735)
Supplement: Supplementary file 1 — Fig. S1. Schematic representation of the genes contained in FUN operon (RoCPR1‐CYP509C12). The sequences of the intergenic regions R1‐R3 are indicated in the table. The sequence of restriction sites is underlined and RBS sequences are indicated in bold. The main restriction sites in the synthetic operon are also indicated. Fig. S2. SDS‐PAGE analysis of the 11α hydroxylating enzymes from R. oryzae in the M. smegmatis recombinant strains. (A) Lanes 1 and 6, molecular mass markers; lane 2, soluble (SN) control extract from MS6039 (pMV261); lane 3, soluble extract from MS6039 (pMFUN); lane 4, insoluble fraction from MS6039 (pMV261) and lane 5, insoluble fraction from MS6039 (pMVFUN). (B) Lanes 1 and 6, molecular mass markers; lane 2, soluble (SN) control extract from MS6039‐5941 (pMV261); lane 3, soluble extract from MS6039‐5941 (pMFUN); lane 4, insoluble fraction (P) from MS6039‐5941 (pMV261) and lane 5, insoluble fraction from MS6039‐5941 (pMVFUN). The bands of CYP and CPR proteins are indicated by asterisks and arrows. Fig. S3. CHO biotransformation by MS6039 (pMVFUN) (FUN) and MS6039 (pMV261) (Control) strains. A) HPLC‐DAD chromatogram (50‐600 nm) (green) and full scan mass spectra (m/z 150‐400) at 0 h and 96 h of growth are shown. B) Magnification of the first 14 min of the HPLC‐DAD chromatogram (50‐600 nm) green, full scan mass spectra (m/z 150‐400), mass spectra of m/z 301 and m/z 303 corresponding to 11αOH‐ADD and 11αOH‐AD are shown Fig. S4. PHYTO biotransformation by MS6039 (pMVFUN) (FUN) strain. HPLC‐DAD chromatogram (50‐600 nm) (green), full scan mass spectra (m/z 150‐400), mass spectra of SITO (m/z 387), CAMP (m/z 383), STIG (m/z 386), 11αOH‐ADD (m/z 301) and ADD (m/z 286) at 96 h are shown. Fig. S5. CHO biotransformation by MS6039‐5941 (pMVFUN) (FUN) and MS6039‐5941 (pMV261) (Control) strains. A) HPLC‐DAD chromatogram (50‐600 nm) (green) and full scan mass spectra (m/z 150‐400) at 0 h and 96 h of growth are shown. B) Magnification of the first 20 min of the [file MBT2-14-2514-s001.pptx]

## Slide 1
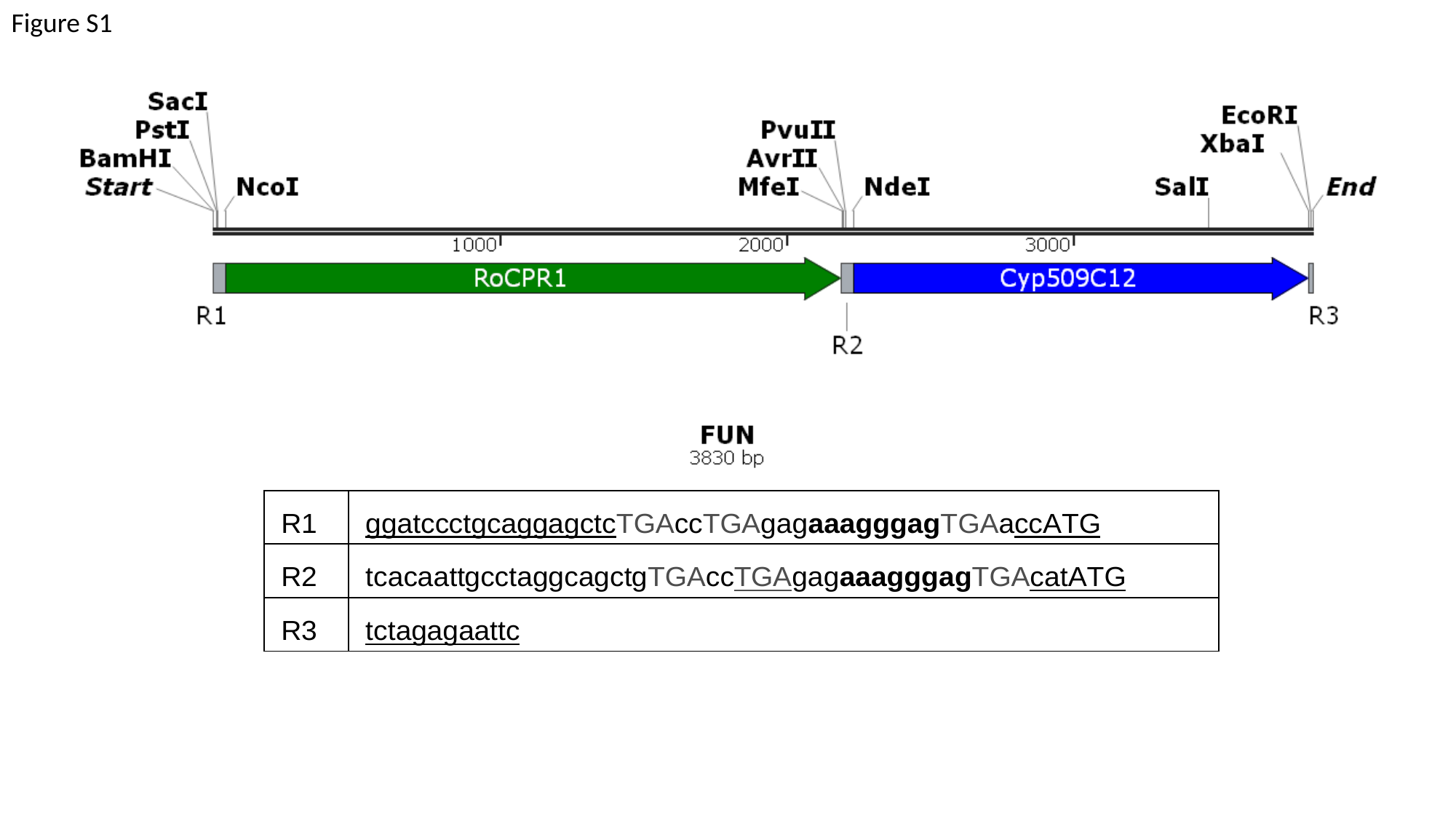

Figure S1

## Slide 2
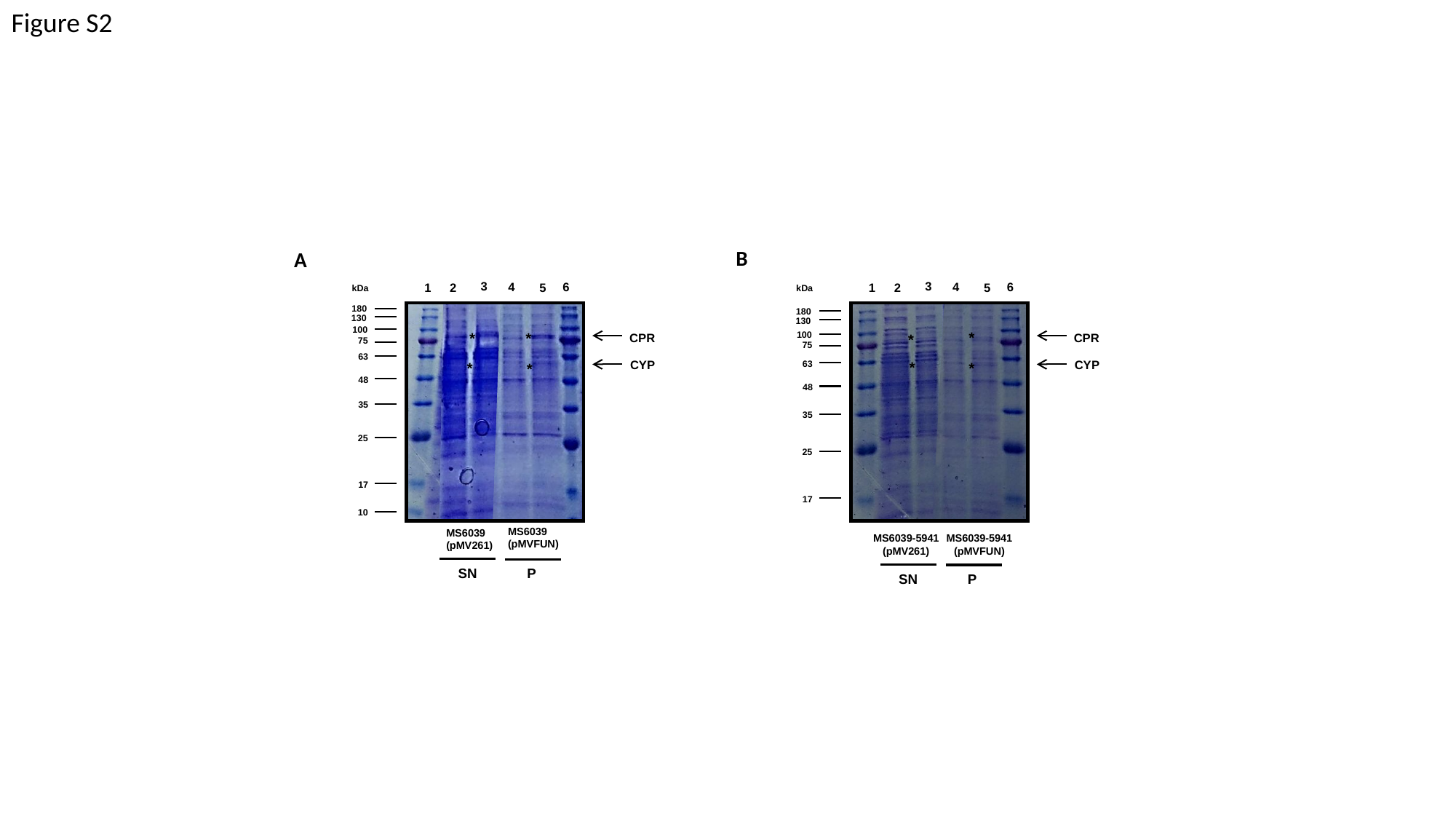

Figure S2
B
A
3
3
4
6
4
6
1
2
5
1
2
5
kDa
kDa
180
180
130
130
100
*
*
*
100
*
CPR
CPR
75
75
63
CYP
CYP
*
63
*
*
*
48
48
35
35
25
25
17
17
10
MS6039 (pMVFUN)
MS6039 (pMV261)
MS6039-5941 (pMVFUN)
MS6039-5941 (pMV261)
SN
P
SN
P

## Slide 3
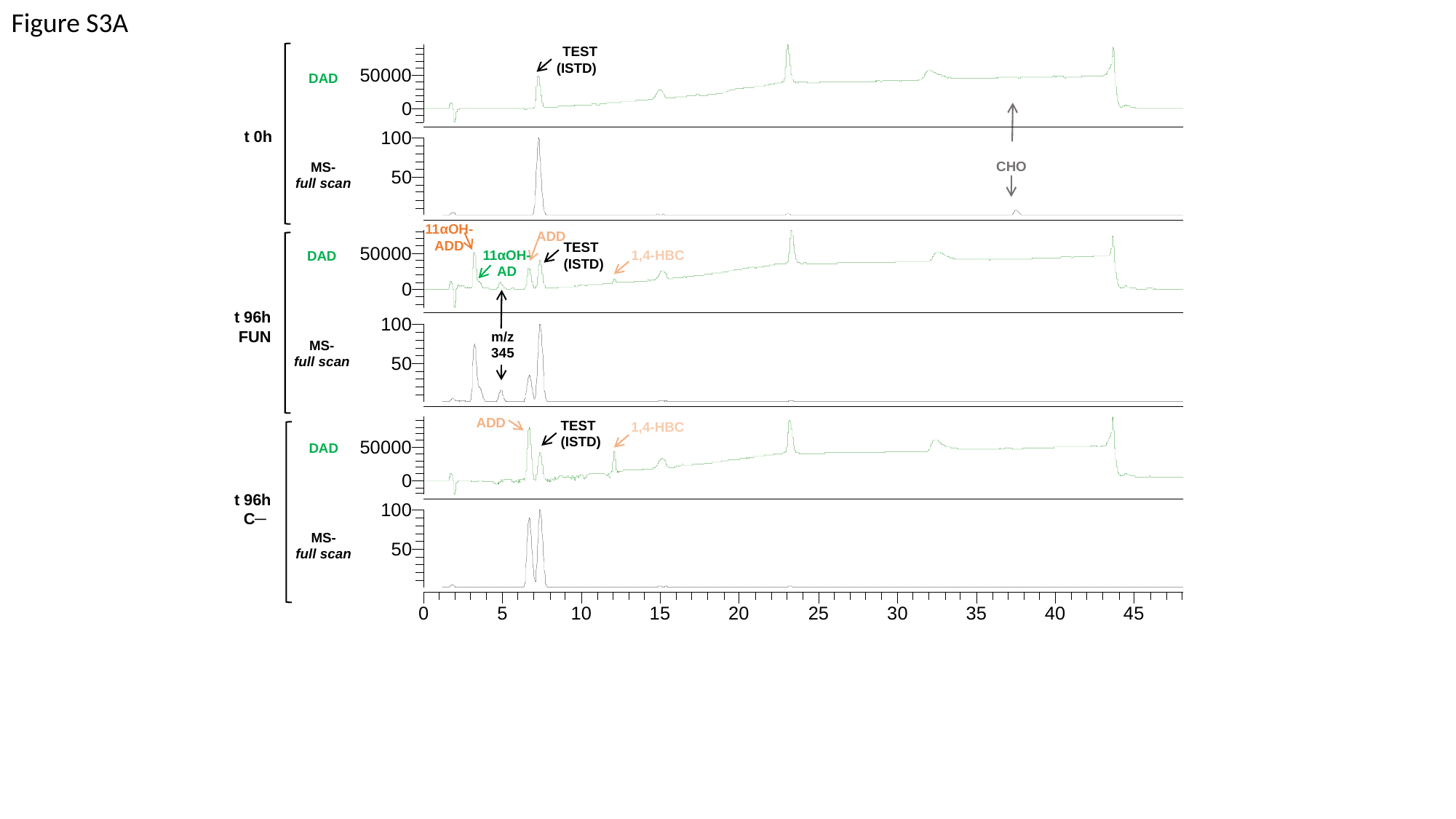

Figure S3A
TEST
(ISTD)
DAD
MS-
full scan
t 0h
CHO
11αOH-ADD
ADD
TEST
(ISTD)
11αOH-AD
1,4-HBC
DAD
MS-
full scan
t 96h
FUN
m/z
345
ADD
TEST
(ISTD)
1,4-HBC
DAD
MS-
full scan
t 96h
C─

## Slide 4
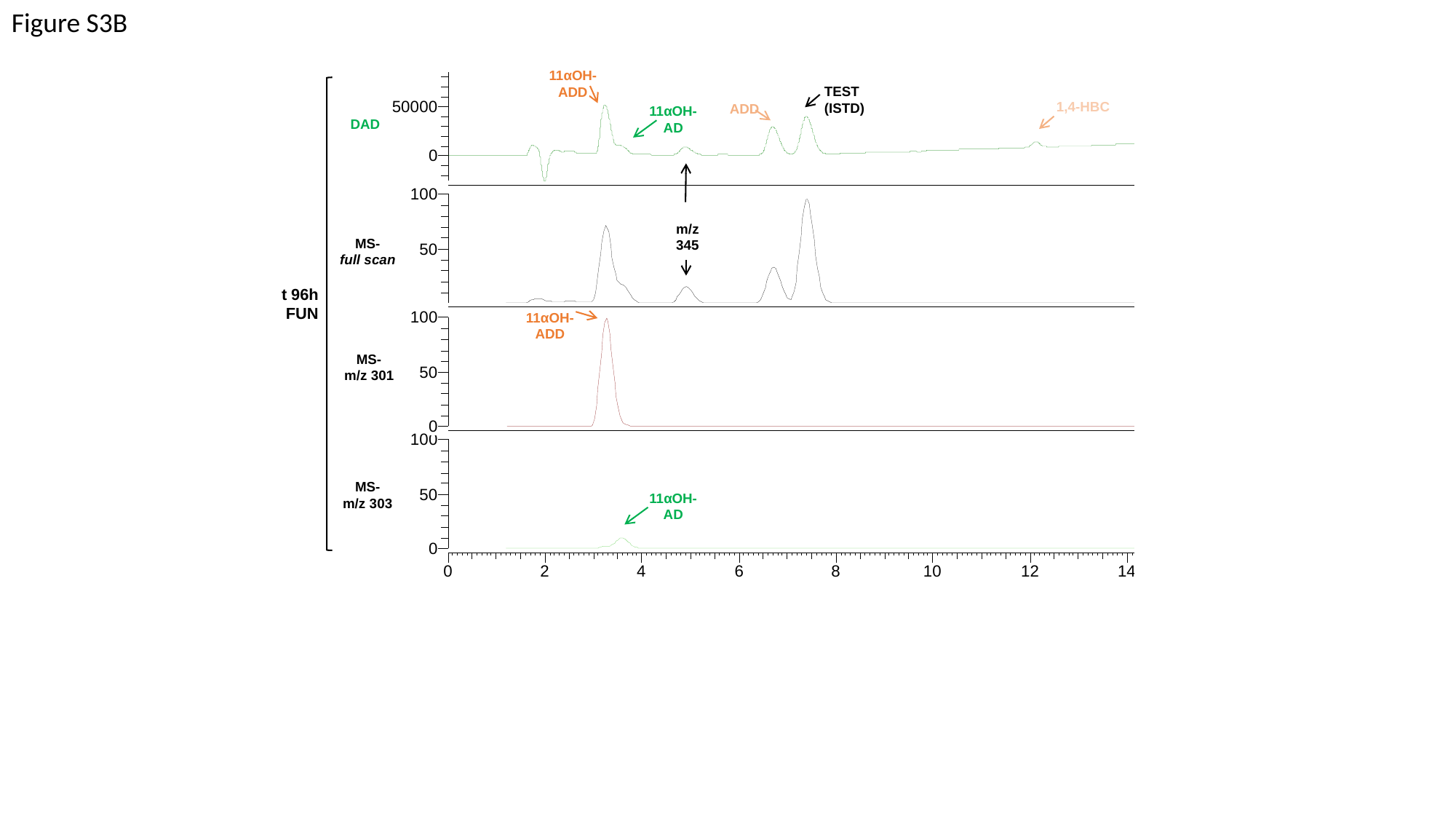

Figure S3B
11αOH-ADD
TEST
(ISTD)
1,4-HBC
ADD
11αOH-AD
DAD
m/z
345
MS-
full scan
t 96h
FUN
11αOH-ADD
MS-
m/z 301
MS-
m/z 303
11αOH-AD

## Slide 5
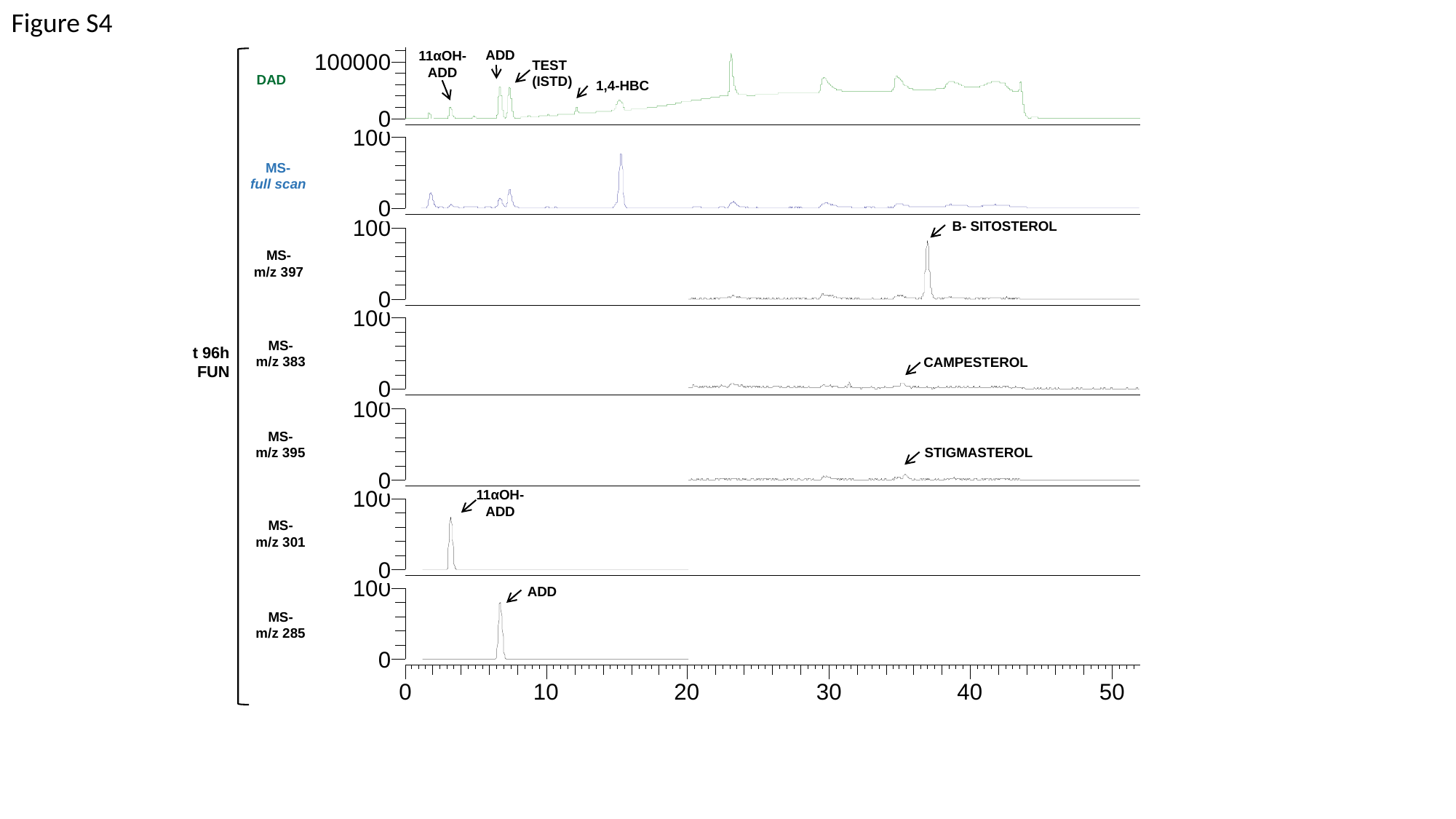

Figure S4
ADD
11αOH-ADD
TEST
(ISTD)
DAD
1,4-HBC
MS-
full scan
Β- SITOSTEROL
MS-
m/z 397
MS-
m/z 383
CAMPESTEROL
MS-
m/z 395
STIGMASTEROL
11αOH-ADD
MS-
m/z 301
ADD
MS-
m/z 285
t 96h
FUN

## Slide 6
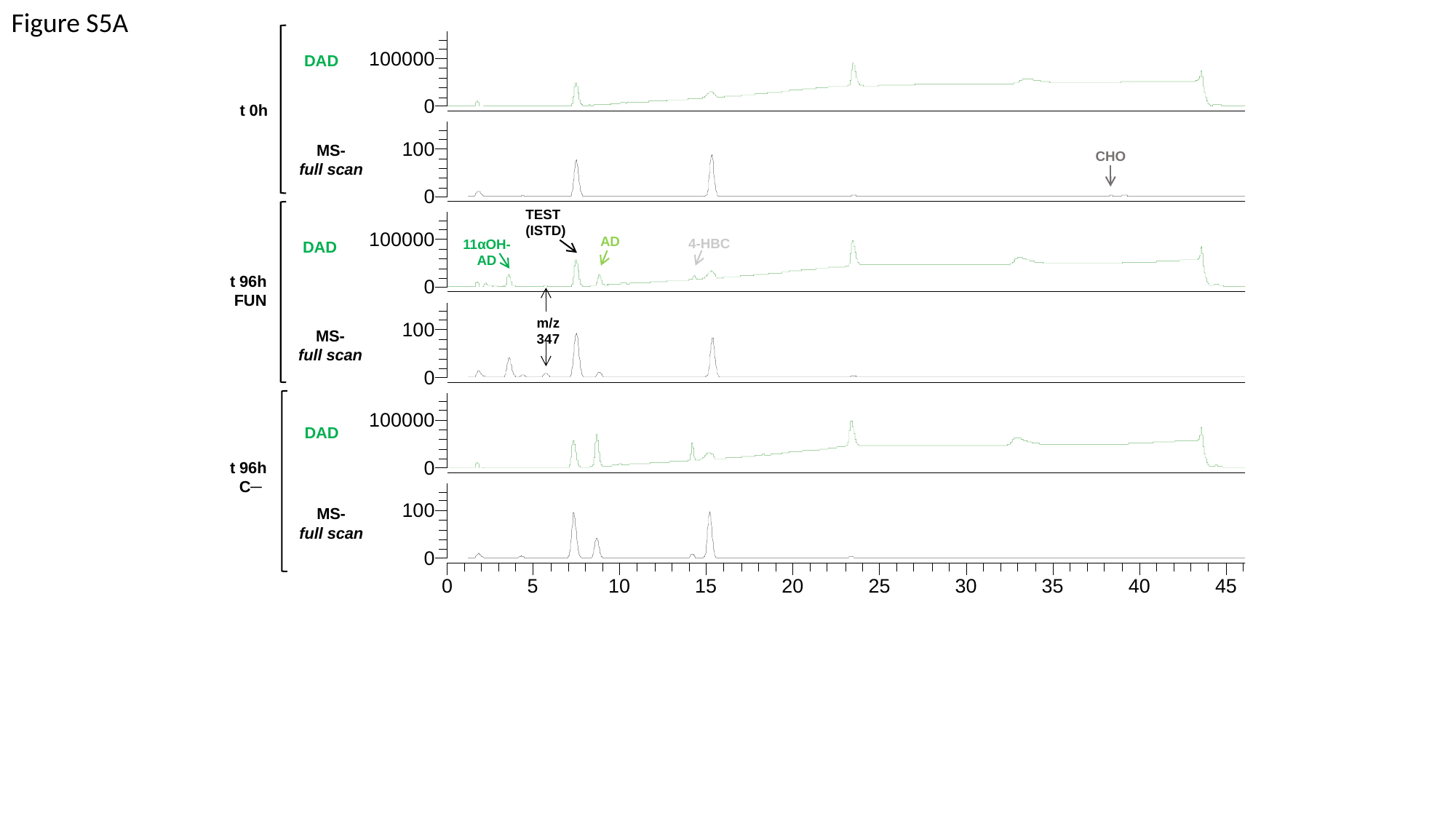

Figure S5A
DAD
t 0h
MS-
full scan
TEST
(ISTD)
AD
4-HBC
11αOH-AD
DAD
t 96h
FUN
m/z
347
MS-
full scan
DAD
t 96h
C─
MS-
full scan
CHO

## Slide 7
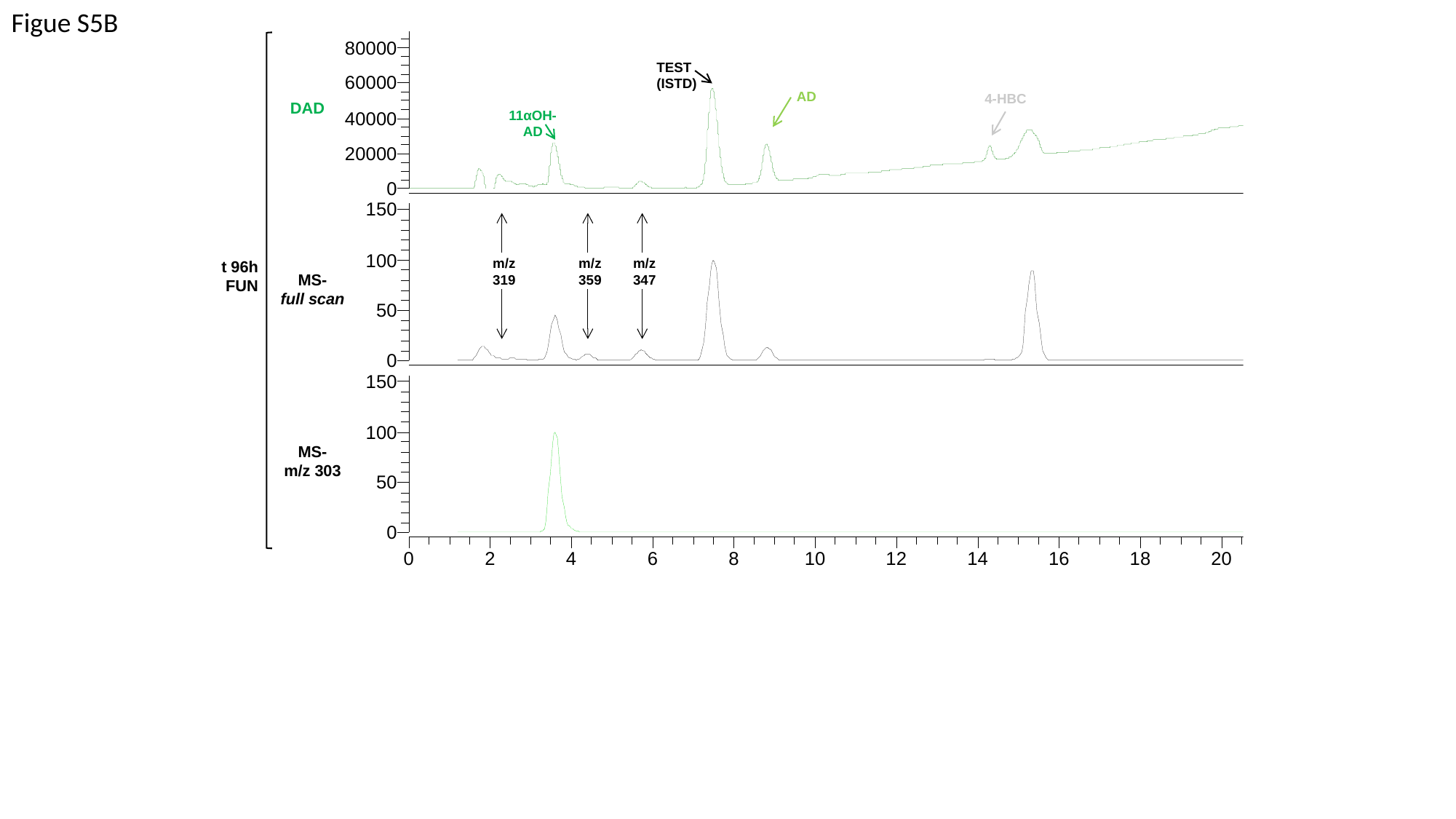

Figue S5B
TEST
(ISTD)
AD
4-HBC
DAD
11αOH-AD
m/z
319
m/z
359
m/z
347
t 96h
FUN
MS-
full scan
MS-
m/z 303

## Slide 8
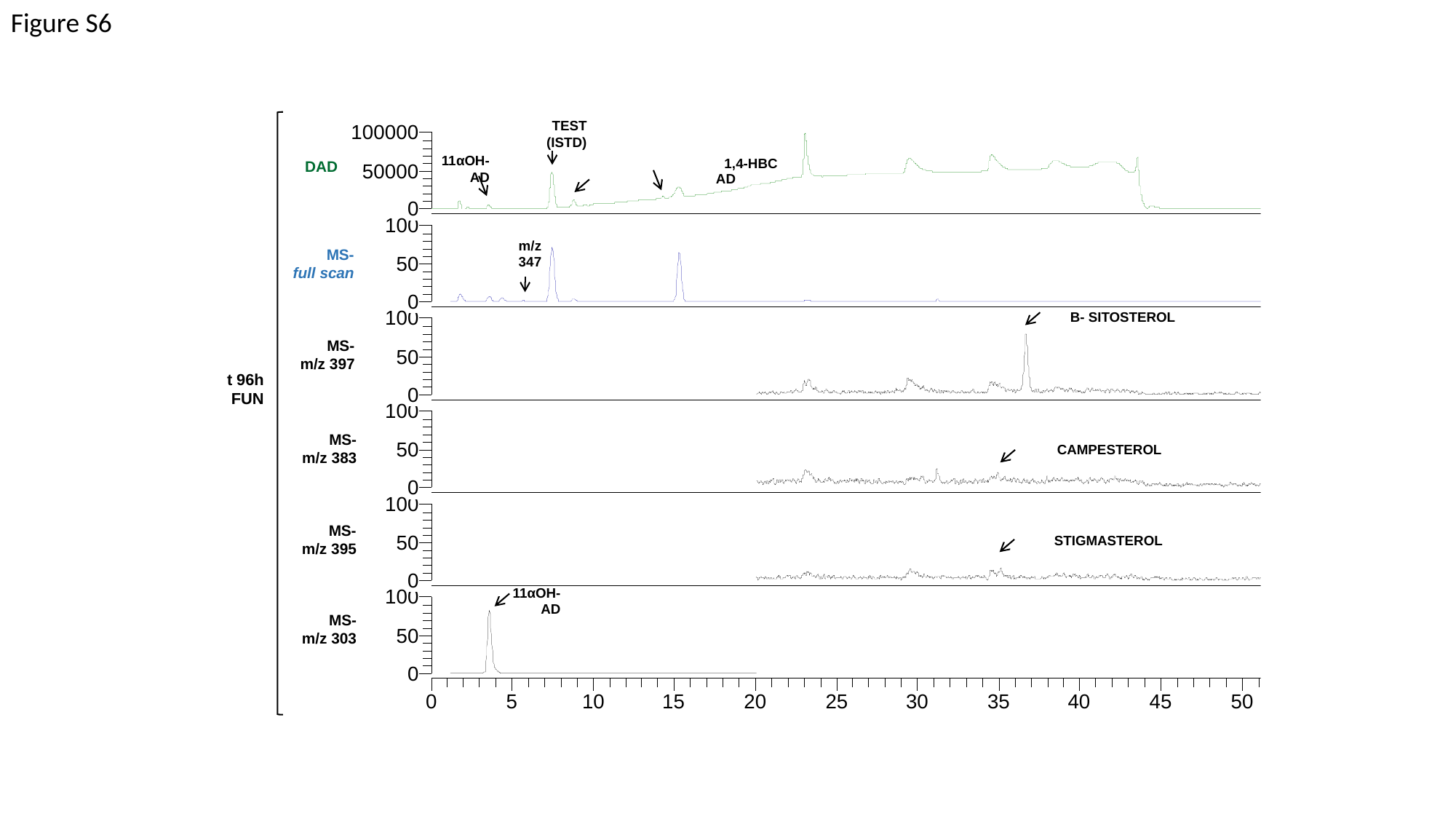

Figure S6
TEST
(ISTD)
11αOH-AD
1,4-HBC
DAD
AD
m/z
347
MS-
full scan
Β- SITOSTEROL
MS-
m/z 397
MS-
m/z 383
CAMPESTEROL
MS-
m/z 395
STIGMASTEROL
11αOH-AD
MS-
m/z 303
t 96h
FUN
